# Natural Language Processing in Large-Scale Neural Models for Medical Screenings

**DOI:** 10.3389/frobt.2019.00062

**Published:** 2019-08-02

**Authors:** Catharina Marie Stille, Trevor Bekolay, Peter Blouw, Bernd J. Kröger

**Affiliations:** ^1^Department for Phoniatrics, Pedaudiology, and Communication Disorders, Medical Faculty RWTH Aachen University, Aachen, Germany; ^2^Applied Brain Research, Waterloo, ON, Canada; ^3^Centre for Theoretical Neuroscience, University of Waterloo, Waterloo, ON, Canada

**Keywords:** neurocomputational model, spiking neural networks, detailed computer simulations of natural language processes, behavioral testing, brain-behavior connection, medical screenings

## Abstract

Many medical screenings used for the diagnosis of neurological, psychological or language and speech disorders access the language and speech processing system. Specifically, patients are asked to fulfill a task (perception) and then requested to give answers verbally or by writing (production). To analyze cognitive or higher-level linguistic impairments or disorders it is thus expected that specific parts of the language and speech processing system of patients are working correctly or that verbal instructions are replaced by pictures (avoiding auditory perception) or oral answers by pointing (avoiding speech articulation). The first goal of this paper is to propose a large-scale neural model which comprises cognitive and lexical levels of the human neural system, and which is able to simulate the human behavior occurring in medical screenings. The second goal of this paper is to relate (microscopic) neural deficits introduced into the model to corresponding (macroscopic) behavioral deficits resulting from the model simulations. The Neural Engineering Framework and the Semantic Pointer Architecture are used to develop the large-scale neural model. Parts of two medical screenings are simulated: (1) a screening of word naming for the detection of developmental problems in lexical storage and lexical retrieval; and (2) a screening of cognitive abilities for the detection of mild cognitive impairment and early dementia. Both screenings include cognitive, language, and speech processing, and for both screenings the same model is simulated with and without neural deficits (physiological case vs. pathological case). While the simulation of both screenings results in the expected normal behavior in the physiological case, the simulations clearly show a deviation of behavior, e.g., an increase in errors in the pathological case. Moreover, specific types of neural dysfunctions resulting from different types of neural defects lead to differences in the type and strength of the observed behavioral deficits.

## Introduction

From the viewpoint of computational neuroscience, a *medical screening* can be seen as a specific task that a subject–or in this case a neural model–has to perform. The result of the task (i.e., the behavior of the subject) is then checked on the basis of a performance scale in order to see whether the subject behaves *pathologically* in accordance with a specific cognitive, language, or speech disorder, or whether the person behaves normally (*physiologically*). The task or scenario involved in a screening is always shaped in a way that the screening is effective for detecting a specific impairment or disorder, here a specific cognitive or a specific language or speech impairment disorder.

Why is it advantageous to simulate a medical screening using a biologically inspired neural model, which is basically a specific sequence of actions to be executed by a patient or here by a biologically inspired neural model? The answer is that the neural deficits leading to neural dysfunctions can be clearly defined in a neural model while also being targeted to specific functional modules of the model. The behavior resulting from a simulation of a model like this can then be analyzed as suggested by the screening. Thus, the simulations done by using the neural model clearly identify the relations between *microscopic neural deficits*—like deficits occurring within neural buffers or on neural connections—and *macroscopic behavioral deficits*. Neural models can accordingly help address longstanding research questions regarding the relationship between low-level properties of neural systems and the high-level behavioral patterns.

To provide context, physiological (i.e., normal) speech processing is described briefly below. Speech processing involves a range of cognitive, motor, and sensory skills that involve numerous neural subsystems. The cognitive system comprises pragmatic, semantic, syntactic and phonological components and interfaces with sensory and motor systems through a phonetic component. The mental lexicon (Dell and O'Seaghdha, 1992; Levelt et al., 1999; Indefrey and Levelt, 2000; Elman, 2004) is an important part of the cognitive system as it serves as a basic knowledge repository for word forms and their meanings. As described in well-established models of language and speech production (Garrett, 1980; Stemberger, 1985; Dell, 1986; Butterworth, 1989; Levelt, 1989; Caramazza, 1997; Dell et al., 1997; Levelt et al., 1999; Indefrey and Levelt, 2004; Indefrey, 2011) the mental lexicon comprises three levels: The concept level stores the meanings of words; the lemma level stores the language-specific grammatical status of words; and the phonological form level stores the sound sequence of words (Friedmann et al., 2013). Conceptual knowledge stored in the mental lexicon can be separated with respect to specific attributes such as size, shape, and color, along with more general category information. For example, dogs and cats belong to the category *animals*, buses and bicycles to the category *objects for transportation*, and so on. A lexical concept is a concept for which a word must exist (Indefrey, 2011). The lemma-level contains lemmata that store grammatical attributes of words, e.g., that *chair* is a *noun* and *singular*. The phonological form level contains phonological and morphological information; for example, it would store the fact that *chair* includes the phonemes /t∫ /, /e/, /ǝ/, and it would also store morpho-phonological variants as well, such as the plural /*chairs*/. For effective processing, lexical entries must contain adequate information within all three levels, and these levels must communicate with each other. Further, within the different levels (conceptual, grammatical, phonological) many associations exist between entries (Brackenbury and Pye, 2005). These associations are intrinsic (Levelt, 1989) and based on similarities at the semantic, grammatical or phonological level. For example, chair and table are linked by the fact that they both belong to the furniture category and are often used together. In language acquisition, children use these relations between words as a basis for developing connections between lexical items (Landau et al., 1998).

Speech processing comprises speech production and speech perception. In both cases, humans need to retrieve information from the mental lexicon. In the case of *speech production*, concepts are activated for the intended utterance, and associated lemmas and phonological forms are subsequently retrieved from the mental lexicon during the process of utterance formulation. Motor plans are then activated and retrieved from a second repository, the *mental syllabary* (Cholin, 2008; Brendel et al., 2011; Kröger and Cao, 2015), which contains sensory and motor information used during speech articulation. It is known that at all levels in the mental lexicon (concept, lemma, and phonological form), more than one item may be activated at one time to different degrees. Consequently, in speech processing a related lexical item can be erroneously named instead of the target item (Indefrey, 2011). In the case of *speech perception*, the mental syllabary and mental lexicon play important roles as basic knowledge repositories both for recognizing syllables and words from the speech signal, and for activating phonological forms, concepts and subsequently the meaning and intention of an utterance.

Language processing is a complex cognitive function and it interacts with attention and memory (Poirier and Shapiro, 2012). For this reason, linguistic testing should not only analyze the nature of the entries and associations of the mental lexicon (e.g., with picture naming), but also verbal short- and long-term memory (to remember and reproduce a certain number of words immediately and after some time), as well as cognitive flexibility through word generation tasks (e.g., name all terms that come to mind about supermarket; Kalbe et al., 2004; Indefrey, 2011).

To analyze the neural basis of speech processing, Indefrey and Levelt (2000, 2004) and Indefrey (2011) performed meta-analysis of imaging studies of the regional cerebral brain activation patterns observed in different lexical tasks. For every stage of speech processing, different activated parts of the brain with fast processing stages could be found. These findings indicate that the model assumptions of speech processing are neurobiologically inspired and therefore, models can be used to derive real-world findings.

This knowledge has already been incorporated into various computer-simulated models in order to verify the assumptions of speech processing and to gain knowledge about pathological language processing. Roelofs (1992, 1997) implemented the theory of a three-level mental lexicon and a spreading activation theory of conceptual driven lemma retrieval in a connectionist computer model (WEAVER). The model produces valid results for naming, object categorization, and word categorization in a picture-word-interference task. Further, semantic facilitation showed valid results in comparison to natural data. Levelt et al. (1999) further presented the computer model WEAVER ++ which analyzes real-time processing in normal word production. The model includes the stages from lexical selection to phonological encoding with access to the mental syllabary.

Another connectionist computer-simulated model has been developed by Dell (1986). It comprises the three levels (conceptual, lemma, and lexeme level) and word retrieval based on intra- and interconnections between these levels. Errors in speech production are modeled by a pathological increase in the rate of decay of primed nodes in the semantic-lexical-phonological network. Two simulated experiments were used to analyze how the theory can be applied to phonological coding processes. The first experiment showed the basic types of phonological errors and their relative frequency. The second experiment was performed to adapt data produced with a technique designed to generate these errors under controlled conditions.

A more biologically inspired computational model of speech acquisition and production is the DIVA model (Guenther, 1995; Guenther et al., 1998, 2006). DIVA is a neural network that models the sensorimotor interactions during articulator control in speech production. The model has been used to analyze behavioral and functional imaging studies of speech processing (e.g., Tourville et al., 2008), and is intended to allow for comparisons between simulated and empirical data. Model simulations produce expectations of acoustic data (e.g., formant frequencies), somatosensory data (e.g., articulator positions), learning rates, and activity levels within specific model components. Further, GODIVA provides an extension for speech production at a suprasegmental level (Bohland et al., 2010).

In summary, in order to cope with the complexity of language processing, the simulation of a linguistic medical screening which includes language processing and verbal short-term memory requires a *large-scale neural model*. This model may include perceptual input processing, motor output processing, and intermediate cognitive processing. Our approach tries to model these components in a biologically inspired way (e.g., Stewart and Eliasmith, 2011; Golosio et al., 2015a,b; Kröger et al., 2016). Large-scale neural models are generally comprised of cognitive processing modules, short-term and long-term memory modules, sensory input, and motor output processing modules, as well as a central executive unit for the temporal coordination of cognitive, perceptual, and motor actions (Stewart et al., 2010a; Eliasmith, 2012; Civier et al., 2013; Golosio et al., 2015a).

One approach, which enables the creation of large-scale neural models, is the Neural Engineering Framework (NEF, Eliasmith and Anderson, 2004; Stewart et al., 2012a). The NEF provides a comprehensive mathematical framework for modeling spiking neurons and neural networks on the basis of three principles: the *representation* of states as neural activation patterns, the *transformation* of neural activation patterns through weighted connections between neurons, and the treatment of neural representations as control-theoretic state variables governed by particular *dynamics*. Neurons are grouped in *neuron ensembles* that represent simple states which can be mathematically represented as scalar or vector values. The representational state of a neuron ensemble is realized by a specific pattern of spiking across all neurons within the ensemble. The semantic pointer architecture (SPA, Eliasmith, 2013; Stewart and Eliasmith, 2014) builds on the NEF to allow the modeling of complex cognitive processes by grouping neural ensembles in sophisticated functional units (Eliasmith, 2012; Eliasmith et al., 2016) controlled by a central executive (Stewart et al., 2010b, 2012a,b). The central executive of a SPA model, here called a *task control module*, is designed to emulate the basal ganglia-thalamus-cortex loop for cognitive action selection (Stewart et al., 2010a, 2012a).

Previous work with large-scale models and speech production revealed insightful results concerning physiological word production and pathological speech in the case of certain speech dysfluencies. Kröger et al. (2016) showed that in a picture naming and halt paradigm (picture presentation is followed by an auditory stop signal for halting speech production) it is possible to build a NEF and SPA model of the neural mechanisms governing self-detection of speech errors in the inner speech loop. The results of this work indicate that speech errors are successfully detected by a monitoring module in the inner speech loop. Furthermore, the model correctly reproduces human behavioral data on the picture naming and halt task. In particular, the halting rate in the production of target words was lower for phonologically similar words than for semantically similar or fully dissimilar distractor words. Senft et al. (2016) simulated a syllable sequencing tasks using a NEF and SPA model of the basal ganglia-thalamus-cortical action selection loop in order to investigate the freezing effect of patients with Parkinson's disease (PD) in more detail. By decreasing the dopamine level parameters in the model's action selection loop by 50%, the freezing effect of patients with PD could be replicated.A further example comes from Civier et al. (2013). They used an impaired form of the computational speech production model GODIVA to examine two hypotheses concerning the neural basis of stuttering: (1) white-matter irregularities interrupt cortico-striatal projections, producing duplicates of executed motor commands; and (2) dopaminergic irregularities interrupt the next syllable's motor program, which leads to dysfluency. The model may also consider the results of brain imaging studies of stuttering.

To evaluate large-scale neural model with respect to its overall cognitive and sensorimotor capabilities, well-established medical screenings are highly useful, since they limit the scope of relevant human actions to a well-defined behavioral scenario. For many screenings, standard data are available with large samples that include both physiological and pathological cases. These data can be used to analyze certain results, either patient-produced or model-simulated. Thus, the tasks defined in medical screenings enable large-scale neural models to be used for the analysis of both normal (or physiological) and pathological behavior of the sort that occurs if a subject suffers from a specific impairment or disorder. In the case of dementia, the DemTect (Calabrese et al., 2004; Kalbe et al., 2004) can be simulated easily, as we show in the following. This specific screening tests cognitive abilities involving speech and language processing. Screenings developed for detecting specific speech and/or language disorder can also be used to examine the language and speech processing components of a neural model (Stille et al., 2017, 2018). Parts of a linguistic test for lexical storage and lexical retrieval in Standard German (WWT 6-10, Glück, 2011) and a screening for the detection of dementia (Calabrese et al., 2004; DemTect, Kalbe et al., 2004) were selected accordingly for simulation in our large-scale neural model.

It is the first goal of this paper to introduce a large-scale model based on the NEF and SPA which is capable of simulating both the WWT and DemTect screening. The second goal of the paper is to simulate parts of both screenings using the normal neural model (i.e., the physiological case) and by using the neural model including neural deficits within different modules of the model (i.e., the pathological case). This allows us to associate deficits in simulated behavior with neural deficits.

## Methods

### Neural Model and the Tested Medical Screenings

Within our computational model we use neural buffers, which are a SPA-specific concept (see Stewart and Eliasmith, 2014). A *neural buffer* consists of D neural ensembles and is able to represent (complex) neural states which can mathematically be represented as a D-dimensional vector of numerical values. The neural activity in a buffer is represented on the one hand mathematically as a D-dimensional vector, and on the other hand as a compressed representation that can be selectively decompressed to retrieve different kinds of cognitive, sensory, and motor information. Semantic pointers can represent cognitive states (e.g., concepts), higher level perceptual states (e.g., complex auditory impressions), or higher-level motor states (e.g., a motor plan for the articulation of a syllable). Thus, a semantic pointer as defined in the SPA is a compressed representation implemented by the activities of a collection of spiking neurons within a neural buffer and is mathematically characterized as a high-dimensional vector (Eliasmith, 2013; Stewart and Eliasmith, 2014). Neural buffers are often non-recurrent and represent neural states which vary quickly in response to different inputs. But neural buffers can also be recurrently connected and then represent *short term memories* capable of holding information for a longer time period.

Neural representations, including semantic pointers, can be transferred from one buffer to another by a simple neural connection. Neural connections between buffers can implement arbitrarily complex associations of the sort that convert representational state “a” activated in buffer “A” to a representational state “b” activated in buffer “B.” Thus, a neural state “a” is now associated with a neural state “b.” To give a concrete example of this process, it is a central task of the mental lexicon to associate the concept of a word (activated in the concept buffer within the production or perception pathway) with the phonological form of the same word (activated in a second buffer within the perception or production pathway, called the phonological form buffer). The neural connection between those two buffers contains knowledge, and in the SPA such connections implement an *associative memory* (Voelker et al., 2014; Crawford et al., 2016). A special subtype of an associative memory is the *cleanup memory* which is needed if the association in buffer B is designed to select the most relevant semantic pointer as a result of a neural association process (Crawford et al., 2016). Other types of connections between buffers may realize the binding of two representational states occurring in buffer A and buffer B, to form structured neural representations of arbitrary complexity. In its most basic form, this binding operation produces an output representational state in some buffer C that encodes the pairing of the representational states in A and B (Eliasmith, 2013).

The representational state of a buffer as a function of time can be visualized by using plots that depict the similarity between the current state of the buffer and other states corresponding to defined semantic pointers (Eliasmith, 2013). To explain, because the neural activity of a buffer can always be characterized as a high-dimensional vector, this vector can be compared to the vectors corresponding to any number of semantic pointers. Taking the dot product between the vector currently represented by the activity in a buffer and the vectors corresponding to a set of semantic pointers will accordingly produce a set of values indicating the degree to which each semantic pointer is activated by the current state of the buffer. This results in a display of all semantic pointers that exhibit some degree of similarity to the pointer represented by the current activity of the buffer (ibid).

Dot products can be computed via the connections between neural ensembles and can also be used to perform action selection by comparing the representational states in particular buffers with states that are associated with particular cognitive actions. Action selection is performed by the task control module (central executive), and within this module all potential actions are coded by semantic pointers; dot products between each of these action pointers with a pointer representing the current state of other neural ensembles allows the selection of the most promising action at a specific point in time. This action is then represented as a semantic pointer that gets encoded in an action control buffer. A detailed description of this process of action selection and action sequencing is given in Stewart et al. (2012a); Eliasmith (2013); Kröger et al. (2016).

Neural models based on the NEF and SPA approach are implemented using a Python based scripting language called Nengo (Bekolay et al., 2014; Sharma et al., 2016). In Nengo, high-level commands are available for configuring and running a neural model, i.e., for defining neuron ensembles, neuron buffers, and all neural connections between ensembles or buffers. The implementation of the task control module (central executive) can be realized easily in this framework by defining all actions in the form of semantic pointers and by providing this information to the neuron ensembles and buffers defining a submodel of the basal ganglia and thalamus (Stewart et al., 2018).

Parts of a linguistic test for lexical storage and lexical retrieval in Standard German (WWT 6-10, Glück, 2011) and a screening for the detection of dementia (DemTect, Calabrese et al., 2004; Kalbe et al., 2004) were selected for simulation in our NEF-SPA large-scale neural model.

The whole WWT (Word Range and Word Retrieval Test for 6- to 10- Year Old Children, Glück, 2011) is a standardized test for: (1) measuring the size of the vocabulary stored in the mental lexicon of a subject; (2) measuring the stability of word naming; (3) measuring the effects of facilitation of word naming by adding semantic and phonological cues; and (4) measuring word comprehension. The test is developed for the Standard German language vocabulary. The standardization was based on test results from 880 German children of appropriate age. Data are available for the WWT naming task, the WWT comprehension task, and task response times. For measuring vocabulary size and stability of word naming, subjects have to twice produce 95 words one after the another on the basis of 95 pictures. These 95 words are mixed with respect to four categories: (1 and 2) 49 nouns or verbs which are presented visually while accompanied by a question given by the instructor (e.g., “what is that?” in case of nouns or “what is he/she/it doing?” in case of verbs); (3) 23 superordinate concepts which have to be named after deducing the appropriate word from four pictures showing objects which belong to one superordinate category and which are accompanied by the question “what are these all?” (e.g., pictures of “chair,” “closet,” “bed,” and “couch” lead to the naming of the superordinate concept “furniture”); and (4) 23 antonyms of adjectives or adverbs (e.g., “old,” “kind”) which express the opposite of a word presented orally by the instructor and embedded in the question “what is the opposite of …?” (e.g., “old” should be named if “new” is said by the instructor). The test must be done four times in direct temporal succession (run R1 to run R4). Within the first and second run (R1 and R2), all 95 target words must be named, while in the third and fourth run only items that were not named correctly in the two preceding runs are queried. In the third run (R3), semantic and phonological cues are given in order to facilitate word naming. These cues can be semantic associations (e.g., “wheelbarrow” is facilitated using the verbally given cue “this is a type of vehicle used to push and transport something, in a garden or on a construction site”) or the cues can also be auditory presentations of the first sounds of the target word as a phonological facilitation (e.g., “the word we are looking for starts with ‘wh…”'). In the last run (R4), word comprehension is measured. After a verbal request, the children should point the mentioned item from a selection of four pictures.

The DemTect (Calabrese et al., 2004; Kalbe et al., 2004) allows the detection of mild cognitive impairments that may occur in early dementia. Five tasks are included: (1) Repetition of a word list (i.e., word repetition task for nouns), (2) transcoding of numbers from digits to text and from text to digits by writing (i.e., number transcoding task, adapted to speaking in our simulations), (3) enumeration of words answering the question “what things can you buy in a supermarket?” (i.e., semantic word fluency task), (4) reverse repetition of two to six digits (i.e., reverse repetition task), and (5) delayed recall of the initial word list of task one (i.e., delayed word repetition task for nouns). The DemTect has been standardized by testing 145 healthy control subjects, 97 patients suffering from a mild cognitive impairment and 121 patients suffering from beginning Alzheimer's disease and thus classified as candidates for mild dementia. The evaluation of the subjects on the basis of the DemTect leads to a clear separation of these three groups (normal with respect to age, mild cognitive impairment, and dementia). The evaluation is based only on the maximum number of points reached by each subject over all five tasks.

**Figure 1:**
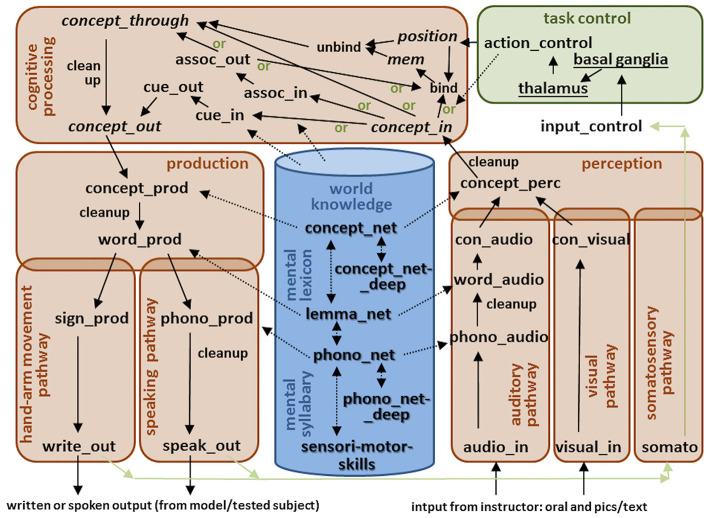
The functional architecture for our large-scale neural model used for two medical screenings (DemTect and WWT). Arrows indicate neural associations between buffers. Buffers within the perception and production pathways allow neural realizations (i.e., neural activation patterns) of S-pointers defined in the mental lexicon and mental syllabary (dashed arrows). S-pointer activity is passed from one buffer to the next within pathways and modules as well as between modules (normal arrows). Short-term memories (recursive buffers) are marked by cursive letters, while all other non-cursive black colored words label non-recursive buffers. Neural associations including cleanup are marked by an extra word attached to the arrow. Different gateways (see green marked word “or”) are controlled by the task control module. The underlined words within the task control module represent specific neural submodules like basal ganglia and thalamus.

The functional architecture for our large-scale neural model used for these medical screenings (DemTect and WWT) is shown in Figure 1. This large-scale model can be subdivided in five main modules: (1) a central executive or task control module; (2) a cognitive processing module including short-term memories; (3) a production pathway including a hand-arm pathway for writing, and a speaking pathway; (4) a perception pathway including an auditory pathway, a visual pathway, and a somatosensory pathway; and (5) a central long-term memory including world knowledge, a mental lexicon and a mental syllabary.

World knowledge includes all acquired concepts and their relations, e.g., a “man” (object) “is a” (relation) “human” (object), or: a “human” (object) “has a” (relation) “nose” (object) (Eliasmith, 2013; Blouw et al., 2016; Crawford et al., 2016; for further information see Kröger et al., 2016). Concepts form the highest layer of the mental lexicon. Each concept is associated with one word which is specified with respect to its phonological form as well as with respect to its grammatical attributes (noun, verb, etc.; gender; number). Lemmata realize the middle layer of the mental lexicon and specify these grammatical attributes while phonological forms realize the lowest layer of the mental lexicon (Levelt et al., 1999). Superordinate concepts (e.g., “days” for concepts like “Monday,” “Tuesday,” etc.; or “months” for concepts like “January,” “February,” etc.) are defined within the deep concept network, and superordinate phonological forms (e.g., for two forms which have the initial sound in common) are defined within the deep phonological network (Kröger et al., 2016). Grammatical knowledge concerning the formation of sentences is still beyond the scope of our current model and thus no grammatical attributes are associated with the lemmata here. However, lemmata are represented by semantic pointers and can be activated in our model in the production pathway as well as in the perception pathway (Figure 1). While the phonological representation is the low-level representation within the mental lexicon, it is also the high-level representation within the mental syllabary. Here, each syllable is associated with a motor plan and an auditory or somatosensory state for syllables (Kröger et al., 2009; Kröger and Cao, 2015), or with motor plans, visual, and somatosensory states for hand-arm gestures (Kröger et al., 2010). The semantic pointers defined here can be activated in neural buffers within the production and perception pathways as well as in the cognitive processing module as is indicated by the dashed lines in Figure 1.

Within the speaking pathway of the production module neural representations of concepts, lemmata, and phonological forms are activated in the neural buffers *concept_prod* via *word_prod* down to *phono_prod*. The further association of phonological forms with motor plans is not implemented in detail in this model. Currently, the speak_out forms are realized as phonological forms. The same is true of the hand-arm movement pathway. This pathway is not implemented in detail in the current version of our model and if writing is demanded in a subtest scenario of the DemTect, it is replaced in our simulations by oral answering. Within the auditory pathway of the perception pathway, concepts, lemmata, and phonological forms can be activated within the neural buffers *phono_audio* via *word_audio* up to *con_audio* (concept activated in auditory pathway). The lower level auditory pathway is not modeled in detail. In our screening scenarios it is thus assumed that the model directly activates the phonological form of an auditory input (*audio_in* is set to the phonological form). The same holds for visual input. Here a visual input (*visual_in*) directly leads to the activation of a concept (e.g., “horse” or “ball” etc.) within the buffer *con_visual* (visually activated concept). Visual and auditory inputs are forwarded to the *concept_perc* (concept perception) buffer which co-activates the semantic knowledge stored in the mental lexicon and in the world knowledge repository (dashed arrow in Figure 1). Because this leads to a co-activation of many associated concepts (e.g., co-activation of “creature,” “man,” women,” etc., if “human” is activated), a cleanup process is introduced as a part of the association from buffer *concept_perc* to buffer *concept_in*, i.e., as a part of forwarding concepts from the perception pathway to cognitive processing. Further cleanup processes are needed for the association from the *concept_prod* to the *word_prod* buffer for the same reason.

The task control module (central executive) selects actions and realizes the sequencing of actions in time. The *input_control* buffer can be seen as a part of the central executive but is shown in Figure 1 as an extra buffer not grouped with any other module, because the temporal activation patterns occurring within the *input_control* buffer are shaped with respect to the temporal structure of the task under execution, and thus represent the priming of the model with respect to the task that is currently being executed. For example, in the case of a word production task (part of WWT), a specific time interval is defined for the presentation of audio and visual inputs (i.e., processing of the acoustic signal produced by the instructor: “Which object do you see in this picture?”). Then, the task control module selects one of the “or”-pathways within the cognitive processing component (the direct pathway from *concept_in* via *concept_through* to the *concept_out* buffer in this case). Later, if the *concept_out* buffer within the cognitive component is activated, the model starts to output a word using the speaking pathway as a part of the production pathway.

In the case of a repetition task (part of DemTect), the task control is more complex. The model forwards the auditory input one after the other to the *concept_in* buffer (listening), then binds each word to its ordinal position (i.e., first word, second word, etc.), and then forwards a semantic pointer representing these binding to a short-term memory (named mem in Figure 1). If this process of listening is completed, a recall process starts which comprises an unbinding of position and concept, and a forwarding of the resulting concept to the *concept_through* and *concept_out* buffer. Thus, the task control module controls the activation of a position buffer as well. The *concept_through* buffer is needed within the cognitive processing module for cases in which a concept is not activated clearly and in which additional cues are used in order to allow a complete activation of that concept in the *concept_out* buffer (semantic and phonological cue task in WWT, e.g., to name the category or an association to facilitate word naming; see above).

Because the sequencing of neural activations from buffer to buffer during cognitive processing and during production (neural associations from buffer to buffer as indicated by arrows in Figure 1)—i.e., because the time interval from activation of *concept_in* to activation of *concept_prod* and further on of *word_prod* and *arite_out* or *speak_out*—can be of different lengths of time, a somatosensory output signal can be used as a control signal for activating the next action within the task control module (e.g., “look at the next picture,” or “listen to the next advice given by the test instructor,” or “produce the next syllable,” cf. Senft et al., 2016, 2018).

The buffers and associations included in the cognitive processing module are shaped with respect to the tasks modeled in this study. This module can be augmented in a straightforward manner to account for further task paradigms. In the case of this study, the cognitive component is shaped with respect to all subtasks of the WWT and the DemTect. In the case of a simple word production task (WWT R1 and R2, without semantic or phonological cues) induced by visual input (e.g., an object on a picture has to be named), the *concept_in* buffer is activated if the model has processed the visual input. The neural activation is directly forwarded to the *concept_through* and *concept_out* buffer. A *concept_through* buffer is included because in the case of additional semantic or phonological cues (WWT, R3), a word candidate may be activated at the level of the *concept_through* buffer but a high level of activation at the *concept_out* level only occurs if additional cues are given via the path defined by the *cue_in* and *cue_out* buffers. The *cue_in* to *cue_out* association (arrow in Figure 1) is fed by specific semantic or phonological knowledge (left dashed arrow from knowledge repository to cognitive processing module in Figure 1), because all words which are associated with a specific phonological or semantic cue need to be activated in *the cue_out* buffer in order to help to activate the correct output. In the case of word production of an opposite or superordinate concept, the pathway via the *assoc_in* and *assoc_out* buffer is used. The association between input and output buffers in this pathway is fed as well by specific world knowledge (right dashed arrow from knowledge repository to cognitive processing module in Figure 1), and the association leads to the activation of an opposite or of a superordinate concept in the *assoc_out* buffer for a specific auditory or visual input.

In the case of memorizing, recalling, and repeating words (DemTect: repetition, reversed repetition, and delayed repetition tasks), words and word positions are bound in a short term memory (mem buffer) and later unbound before the words are (re-)produced by the model (see binding and unbinding buffers within the cognitive processing module in Figure 1). In the case of the DemTect semantic word fluency task, which involves producing a sequence of words based on a prompt, the output within the association pathway (*assoc_out* buffer) needs to be stored in short-term memory in order to prevent the repetition of already mentioned words. The DemTect number transcoding task can be solved by using the association pathway in tandem with the binding and unbinding pathway.

The complete large-scale model comprising all tasks for both screenings includes about 25 buffers and about 30 associative memories of which five are cleanup memories. The semantic pointers are of 64 dimensions. Thus, each neuron buffer includes 64 ensembles and each ensemble consists of 50 neurons leading to 64 × 50 = 3,250 neurons per buffer. Because associative memories contain twice as many neurons as buffers, the model consists of 25 × 3,250 = 81250 neurons for buffers and 30 × 6,500 = 195,000 neurons for associative memories including the modeling of cleanup, binding, and unbinding processes. Each neuron ensemble within basal ganglia and thalamus comprise 50 model neurons leading to 2,100 neurons in basal ganglia and 400 neurons in thalamus. The large-scale model is implemented in Nengo (Bekolay et al., 2014) using default leaky integrate and fire neuron model. All parameters concerning neurons and neural connections are set to the Nengo default values. Simulations were run on a normal desktop computer and took about 5 min for the simulation of one subtask for one item (thus 1 h and 40 min for the simulation of 20 items in run 1 of WWT), and about 30 min for the simulation of one subtask of DemTect (10 words).

The knowledge repository used for modeling DemTect as well as WWT comprises about 1,000 concepts comprising the *concept_net* (Figure 1) and about 250 superordinate concepts comprising the *concept_net_deep*. Thus, about 1,000 lemmata and about 1,000 phonological forms are included and the phonological forms can be split into about 1,200 syllabic and subsyllabic parts (syllable initial and syllable final consonant clusters and single sounds) comprising the *phono_net_deep* (Figure 1).

### Simulations

Two simulation experiments were performed. One experiment involves a part of the WWT (picture induced word naming) and one experiment involves a part of the DemTect (repetition of a word list).

Simulations were done using the normal neural model (physiological case) and using the neural model modified by neural defects like ablation of the number of functioning neurons in a buffer or in an associative memory. This introduces neural dysfunctions with respect to the representations in specific buffers or with respect to the associations implemented by connections between buffers.

To simulate neural dysfunction in the neural network, we used an ablate function which is implemented in Nengo (Bekolay et al., 2014). This function silences a specific percentage of randomly selected neurons within a chosen neural buffer. Ablating 0% of the buffer leaves it unchanged, while ablating 100% of the buffer silences it entirely, meaning that no neurons in the buffer will ever have any activity.

Ablations can be interpreted as neural dysfunctions that lead to a partial disruption of the normal behavior of the respective buffer. The ablate function works on normal neural buffers as well as on associative memories (which themselves are neural buffers). For associative memories, ablation is analogous to disrupting the connections between buffers. Also, multiple buffers and several associative memories can be ablated at the same time. However, to build a direct connection between the neural dysfunction and the high-level behavioral output, it is advisable to disrupt the buffer and associative buffer individually. Neurophysiological findings, however, do not rule out that in pathological cases multiple buffers and connections are disturbed. An isolated disorder is rather rare.

We simulate picture induced word naming for 20 nouns included in the WWT. This is a simulation of a part of R1 of the WWT and called *reduced R1 of WWT*. Simulations are done using the normal model and using a model in which a specific percentage of neurons are ablated in the concept production buffer (*concept_prod* in Figure 1) and in the concept-to-word production associative cleanup memory (arrow between *concept_prod* and *word_prod* in Figure 1).

We have chosen these ablations in keeping with the literature on semantic-lexical developmental disorders. There are two hypotheses of the underlying functional deficit in semantic-lexical disorders, the *storage* hypothesis and the *retrieval* hypothesis. Some researchers argue that word finding deficits reflect underdeveloped *storage* of words and their meaning in the mental lexicon (e.g., Kail and Leonard, 1986; McGregor and Windsor, 1996). Well-developed word storage results in permanent and complete entries (German, 2015). Children with word finding deficits, according to storage hypothesis, may have acquired fewer words, or have poorly organized conceptual storage (McGregor and Appel, 2002; Gray, 2005; Seiger-Gardner and Schwartz, 2008). This can be seen in poorer naming performance by children with word finding disorders (Messer and Dockrell, 2013). The *retrieval hypothesis* states that the mental lexicon is comparable to that of a normally developed child, but lexical processing (i.e., word retrieval) is less efficient (Fried-Oken, 1987; Newman and German, 2002). This hypothesis is supported by the fact that children with WFDs appear to have momentary disruptions in retrieving a known word from the lexicon (Gershkoff-Stowe and Smith, 1997), and by the fact that retrieval errors are non-systematic and transient (Gershkoff-Stowe, 2002).

Based on this prior research, we ablated the concept buffer in accordance with the storage hypothesis and the concept-to-word buffer in accordance with the retrieval hypothesis. These both buffers are most sensitive for word processing and forwarding within our computational model.

**Figure 2:**
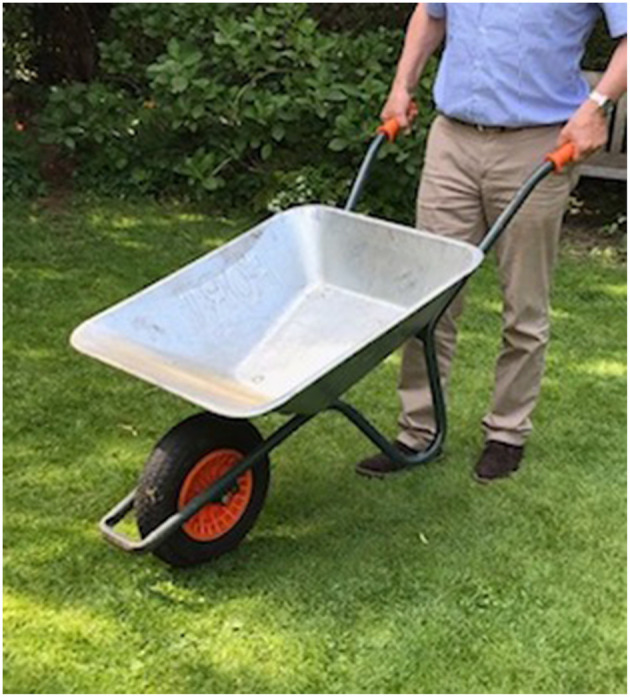
Exemplary replication of a WWT 6–10 image (Glück, 2011) which serves as basis for the word naming task.

The simulation for picture induced word naming is performed as follows, for the example of naming the word “wheelbarrow.” The descriptions can be understood through reference to Figure 1. Following is a running through example given for naming the word “wheelbarrow,” divided into the individual processing steps. Figure 2 shows a replicated photo, which is similar to a picture from WWT and is supposed to elicit the word “wheelbarrow.”

1. Four visual inputs as target items activate the *visual_in buffer* within the visual pathway. These different visual inputs are given because the model may not directly focus on the target object (i.e., the wheelbarrow), as opposed to other objects. Examples of other objects in this case include the “grass lawn” on which the wheelbarrow is placed, the “man” who is pushing the wheelbarrow, and parts of the wheelbarrow like the “wheel” (cf. Figure 2). Many other semantic pointers are activated in the *visual_in* buffer to varying degrees. This results from the fact that about 1,000 words were coded with respect to their visual and auditory input forms, their phonological forms (*phono_audio* and *phono_prod*), as lemmata (*word_audio* and *word_prod*), and as concepts (all buffers within the cognitive processing module and all concept buffers within perceptual and production pathway). These pointers are activated partially both because all the pointers defined in the 64-dimensional vector space are not totally orthogonal from one another, and because semantic or phonological relations exist between different semantic pointers which may affect other related semantic pointers as well. Thus, a cleanup memory is used to map the most relevant semantic pointer from the concept perception buffer to the concept input buffer.
  Because this task is focused on speech production deficits with respect to the naming of visually recognized objects, the perceptual side of the task is simplified that the last object fed into the visual pathway of the model is the target object to be named. Therefore, the visual input “wheelbarrow” will be passed on through *concept_visual* and *concept_perception*. In the concept perception buffer other concepts are strongly co-activated because of access to the mental lexicon. Thus, a cleanup memory is used to map the most relevant semantic pointer from the concept perception buffer to the concept input buffer (*concept_perc* and *concept_in* in Figure 1).
2. Simultaneously, semantic pointers activated in the input control buffer define and separate the time interval of auditory and visual perception, labeled as Q_NOMEN (question for noun: “Which object do you see in the picture?”). This time interval is followed by the time interval of word production, labeled as PRODUCE_NOMEN. The action control buffer activates the action pointer PROCESS_NOMEN followed by SPEAK for these two-time intervals.
3. Within the cognitive processing system, the concept input buffer *(concept_in* in Figure 1) is activated by the semantic pointer “wheelbarrow” which is forwarded to the concept through, concept output, and concept production buffers (*concept_through, concept_out, concept_prod* in Figure 1).
4. In the concept production buffer, other concepts are strongly co-activated because of access to the mental lexicon. Thus, a cleanup memory is used to map the most relevant semantic pointer from concept production buffer to word production buffer on the output side.
5. The word activation occurring in the word output buffer then is associated with the corresponding phonological form activated in the phonological form buffer (*phono_prod* in Figure 1) on the production side of the model. Both buffers have access to the knowledge of the mental lexicon and mental syllabary.

The repetition of ten nouns is part of the first subtask of DemTect and has been simulated using the normal model as well as a model in which a specific percentage of neurons are ablated in the concept input buffer (*concept_in*, Figure 1), in the associative memory between concept through buffer and the concept output buffer (arrow between *concept_through* and *concept_out*, Figure 1), and in the memory buffer (mem, Figure 1).

We have chosen these ablations to simulate short-term memory impairments within the cognitive processing. Impairments of short-term memory are the core symptom of mild cognitive impairments (Petersen et al., 1999, 2001a,b) and the most prominent impairment of dementia (American Psychiatric Association, 1996), specifically in Alzheimer's disease (NINCDS-ADRDA criteria, McKhann et al., 1984; Kalbe et al., 2004). A wordlist with immediate recall is a well-established paradigm to test memory (Kalbe et al., 2004). We accordingly ablated the short-term memory buffer, which are responsible for memorizing concepts, concept input buffer which refers to the ability to keep information and bind with the required position and the *concept_through* to *concept_out* buffer which refers to the ability to forward this information. We have chosen these buffers because the memory and recall processes seem to be the most sensitive processes in our computational model.

The simulation for repetition of a word list is performed as follows. The descriptions can be understood with reference to Figure 1. Following is a running through example given for repetition of a word list, divided into the individual processing steps.

1. The words produced by the test instructor and fed to the auditory input buffer of the model are “plate,” “dog,” “lamp,” “letter,” “apple,” “pants,” “table,” “lawn,” “drinking glass,” and “tree.” These words are forwarded to the phono audio buffer (*phono_audio* in Figure 1) and converted into phonological forms. These phonological forms are then forwarded and converted into lemmata in the word audio buffer, before being forwarded and converted into concepts in the concept audio buffer (cf. Figure 1).
2. Simultaneously, the temporal succession of actions occurring in the task is predefined in the *input_control* buffer. In the case of the repetition of a word list, semantic pointers are defined for the *input_control* action “keep the following words in your mind” (semantic pointer: KEEP); followed by semantic pointers stating: “listen to item 01,” “listen to item 02,” …, “listen to item 10” (semantic pointer: LISTEN_I01,…, LISTEN_I10); followed by a semantic pointer defining the switch to “now try to repeat as many of the 10 words as you can” (semantic pointer: RECALL); followed by 10 semantic pointers “try to reproduce item 01 to item 10” (semantic pointer: PRODUCE_I01,…, PRODUCE_I10).
  The semantic pointers activated in the action control buffer define the two main time intervals of this subtask of DemTect, i.e., KEEP and RECALL. During the time interval defined by KEEP, the audio input pointers are associated with semantic pointers for concepts activated in the concept audio buffer and forwarded to the concept perception buffer.
3. The activated semantic pointer is then forwarded to the *concept_in* buffer. As can be seen from Figure 1, a binding of the neural activation occurring in the concept buffer with the neural activation occurring in the position buffer is then carried out in the model and forwarded to the memory buffer (bind and mem in Figure 1). The semantic pointers occurring in the position buffer (position in Figure 1) are Pos_01 to Pos_10 and are activated by the task control module. These semantic pointers occur in temporal synchrony with the semantic pointers for the concepts of the 10 input words. The results of the binding process are 10 new semantic pointers, i.e., bindings of position pointers and concepts, labeled as P01_Teller, to P10_Baum. These bindings are stored in the short-term memory buffer (mem in Figure 1 including short-term submemories not shown in the figure).
4. During the RECALL time interval, all position pointers are activated one after the other again for a second time within the position buffer and an unbinding process takes place using the representation in the position buffer and the representation in the memory (see unbind in Figure 1). The resulting neural representation is forwarded to the concept through buffer, while exhibiting similarity to the semantic pointers for concepts. Finally, during forwarding of these neural activation patterns to the concept output buffer, a cleanup associative memory is used in order to clearly select a most strongly activated semantic pointer at each point in time.
5. At the level of the concept output buffer a sequence of semantic pointers appears which is further forwarded to the concept production buffer and to further buffers within the speaking pathway (Figure 1).

### Source Code

The respective source code for the simulation of the picture induced word naming (20 nouns) from the WWT and for the simulation of the word list repetition from the DemTect is provided as additional material. The source code for the picture induced word naming (20 nouns) is labeled as WWT_without_cues.ipynb. The source code for the repetition of a word list is labeled as DemTect_repetition.ipynb. Simulations were done using these ipython notebooks within an anaconda3 python environment.

## Results

### Picture Induced Word Naming

The *picture induced word naming task* (R1 of WWT, reduced to 20 nouns) was simulated three times using the normal model and three times for each of the two pathological cases including different degrees of ablation (0 to 90%).

**Figure 3:**
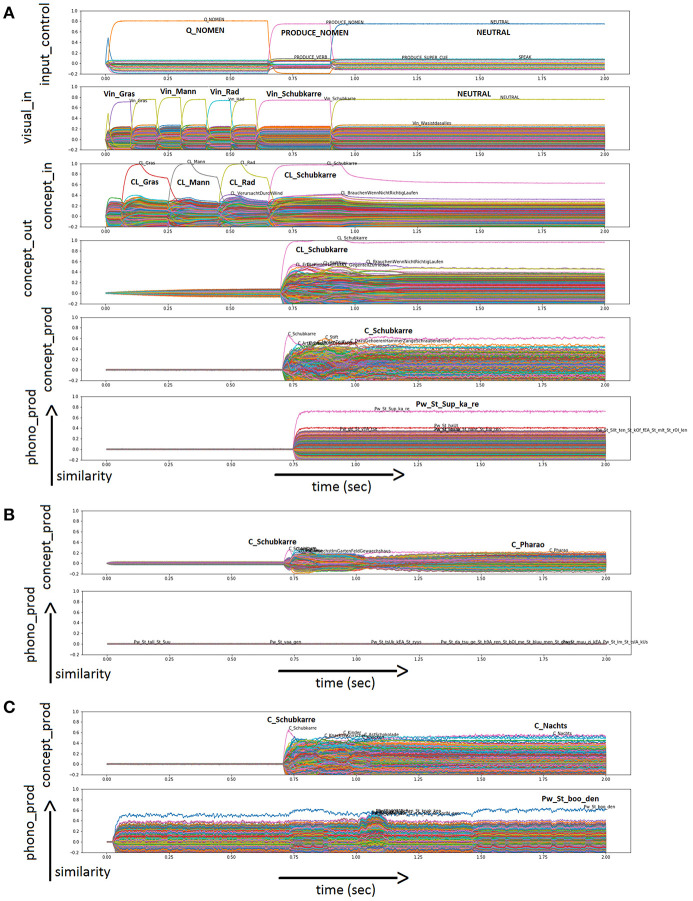
**(A)** Similarity values of S-pointer activation occurring in different neuron buffers over time during simulation of a picture naming task of “Schubkarre” (“wheelbarrow”) in the physiological case. Rows indicate neural similarity values of different neural state buffers of our neural model over time (t). Each S-pointer similarity value over time is represented by a trajectory with specific color. A similarity value of an S-pointer at a point in time is the dot-product of that S-pointer with the unity vector in the direction of the most active S-pointer at that point in time. The number of colors is limited, so the same color may occur for different S-pointers. The height of the graph shows the amount of activation. All other buffers defined in the model are present but not shown in this figure for clarity. In the *Phonological Production Buffer*, the target word is displayed in a phonetic form with the stressed syllable (phonetic transcription with SAMPA, 2005). In row five, words are overlapped as the activation level is very similar. These are co-activation within the word corpus of our model. These items are linked by semantic or associative links to the target item. Furthermore, there are similarity plots for semantic pointers activated in different output buffers of the neural model for the naming of “Schubkarre” (“wheelbarrow”) in the cases of sample run with **(B)** 60% ablation for the neurons within the concept production buffer; and **(C)** 80% ablation for the neurons within the associative memory, realizing the neural association from concept to word production buffer. Please use the input buffers of **(A)** because of the same task and input.

In the physiological case, all 20 nouns are named correctly in all runs. Figure 3A shows an example of physiological word processing with respect to image naming for “wheelbarrow” (German: “Schubkarre”). In this simulation, the task is “PRODUCE_NOMEN” (see *Input Control* buffer). The target word is “Schubkarre” (“wheelbarrow”). Additional visual concepts occurring as pictures at the WWT test sheet for “Schubkarre” (“wheelbarrow”) are activated as well for brief time periods: “Gras” (“grass”); “Mann” (“man”); “Rad” (“wheel”; see *Visual Input* Buffer). The action control buffer (not shown in Figure 3) indicates that the word is produced (S-pointer “SPEAK”) after processing the input (S-pointer “PROCESS_NOMEN”). The production of the word is displayed in the *Conceptual Production Buffer, Word Production Buffer* (not shown in Figure 3), and *Phonological Production Buffer*. It can be seen that “wheelbarrow” is passed on reliably from buffer to buffer. In the *Conceptual Production Buffer*, many other entries are also activated. This is the result of semantic and associative connections in the mental lexicon.

**Figure 4:**
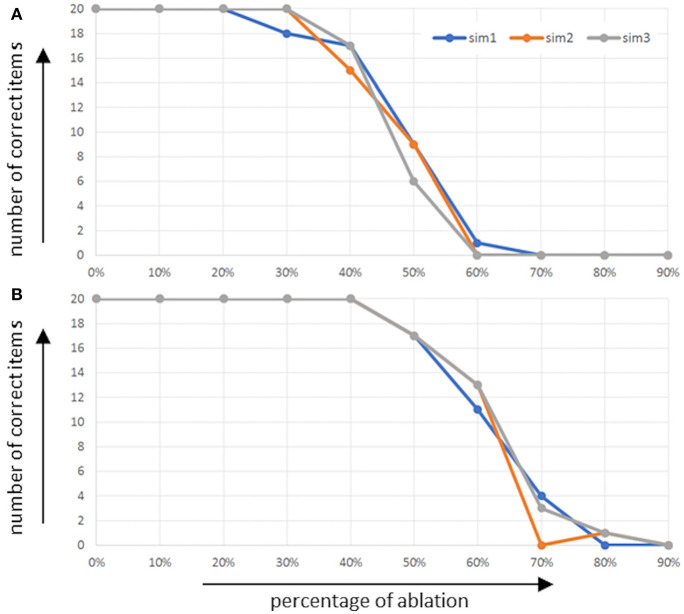
Number of correctly named words (number of correct items) as function of percentage of ablated neurons **(A)** within the concept production buffer and **(B)** within the concept-to-word clean up associative memory.

The overall performance of the model in the picture naming task in terms of the number of correctly named words is given in Figure 4 as a function of the percentage of ablated neurons within the respective buffers [i.e., the concept production buffer (Figure 4A) and the concept-to-word clean up associative memory (Figure 4B)]. It should be kept in mind that an associative memory which connects between two buffers itself is a buffer.

It can be seen that in the case of ablation in the concept production buffer, no effect occurs up to about 30% ablation, and that the performance of correctly named items decreases down to zero until about 60% of neurons are ablated. In the case of ablation of the associative memory, the effect starts and ends later. It starts at about 50% ablation of all neurons in this memory and ends at about 80% of all neurons.

Figure 3 shows two more cases, as an example of incorrect output. Only the *Conceptual Production Buffer* and *Phonological Production Buffer* (Figures 3B,C) are shown as output, since the input is identical to Figure 3A. Figure 3B shows the case of 60% ablation in the concept production buffer. It can be seen that the activation level of semantic pointers (e.g., the semantic pointer representing the correct concept C_Schubkarre) is already strongly reduced in comparison to the normal case (see *Conceptual Production Buffer* in Figure 3B compared to Figure 3A). In addition, many other concepts are activated as well (see *Conceptual Production Buffer*). In the case of the example displayed in Figure 3B, this results in no word activation after the cleanup. Furthermore, no semantic pointer is activated at the level of the phonological form (see *Phonological Production Buffer*).

Figure 3C shows the case of 80% ablation in the clean-up associative memory between the concept production buffer and the word production buffer. It can be seen that the semantic pointer of the correct concept C_Schubkarre is still the most strongly activated concept in the concept production buffer (Figure 3C), although other entries are also strongly activated. However, the correct item cannot be passed on because of the strong activation of other items. Therefore, the target concept is no longer the most strongly activated concept in the word form buffer (not shown in Figure 3C) and in the phonological form buffer within the speech production pathway (Figure 3C). A different (wrong) word is produced in the case of the displayed example (Figure 3C).

The experimental data (standard data) of WWT for the corresponding age category [7;6 to 7;11 (years;month)] and nouns (95 children from the experimental group of standard data ascertainment of WWT 6-10; Glück, 2011) revealed that the number of 11 correctly named items is shown as a cut-off value. Fifty percent of the children within the experimental group have shown a performance (number of correctly named items) over 11 and 50% of the children under 11. This evaluation provides information on whether children show age-appropriate results or pathological ones. This is derived from the standard-normal distribution. Eleven correctly named items is the median, and within the standard deviation (SD) ± 1 the children show normal age-related performance.

### Repetition of a Word List

The first subtask of DemTect, i.e., *repetition of a word list*, was simulated three times using the normal model and three times for each of the two pathological cases including different degrees of ablation (0–90%).

**Figure 5:**
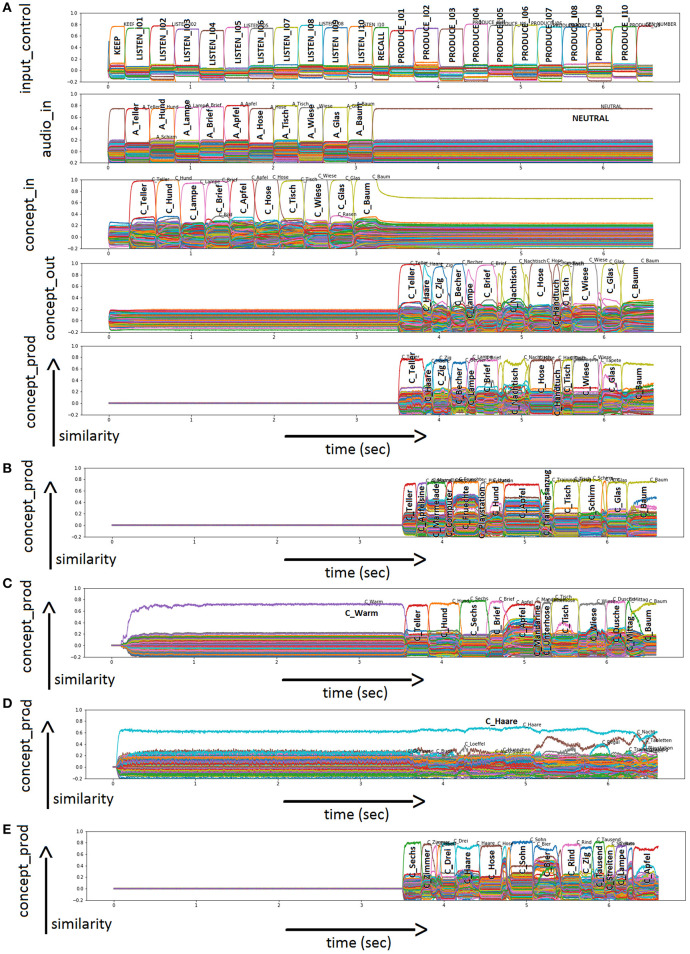
**(A)** Similarity plot for semantic pointers activated in different buffers of the neural model for word repetition (subtask 1 of DemTect) in the physiological case. Rows indicate neural similarity values of different neural state buffers over time (t). Each S-pointer similarity value over time is represented by a trajectory with specific color. A similarity value of an S-pointer at a point in time is the dot-product of that S-pointer with the unity vector in the direction of the most active S-pointer at that point in time. The number of colors is limited, so the same color may occur for different S-pointers. The height of the graph shows the amount of activation. All other buffers defined in the model are present but not shown in this figure for clarity. Furthermore, there are similarity plots for semantic pointers activated in the *Conceptual Production Buffer* of the neural model for word repetition (subtask 1 of DemTect) in the cases of a sample run with **(B)** 30% ablation for the neurons within the conceptual input buffer; **(C)** 30% ablation for the neurons within the associative memory associating the concept through and concept output buffer; **(D)** 50% ablation for the neurons within the associative memory associating the concept through and concept output buffer; and **(E)** 3% ablation for the neurons within the memory buffer (mem). Please use also **(A)** because of the same task and auditory input.

In the physiological case, the repetition of ten nouns is the first subtask of DemTect and has been simulated using the normal model as shown in Figure 5A. In this simulation, the task is “LISTEN” and “PRODUCE” in the given order (see *Input Control Buffer*). The target words are “Teller” (“plate”), “Hund” (“dog”), “Lampe” (“lamp”), “Brief” (“letter”), “Apfel” (“apple”), “Hose” (“pants”), “Tisch” (“table”), “Wiese” (“lawn”), “Glas” (“drinking glass”), and “Baum” (“tree”) presented as auditory input (see *Audio Input Buffer*). The action control buffer indicates that the words are kept (“KEEP”) and to be named (“RECALL”) (not shown in Figure 5A). In the buffer *concept_through*, many other entries are also activated. A cleanup memory is used to map the most relevant semantic pointer from the *concept_through* buffer to the *concept_out* buffer (not shown in Figure 5A). The production of the words is displayed in the *Conceptual Production Buffer*. For the sample simulation displayed in Figure 5A, the model was able to repeat eight out of ten items correctly (see *Conceptual Production Buffer*). The remaining semantic pointers occurring in the concept output or concept production buffers represent other words, not belonging to the list of the ten target words (i.e., incorrect items).

**Figure 6:**
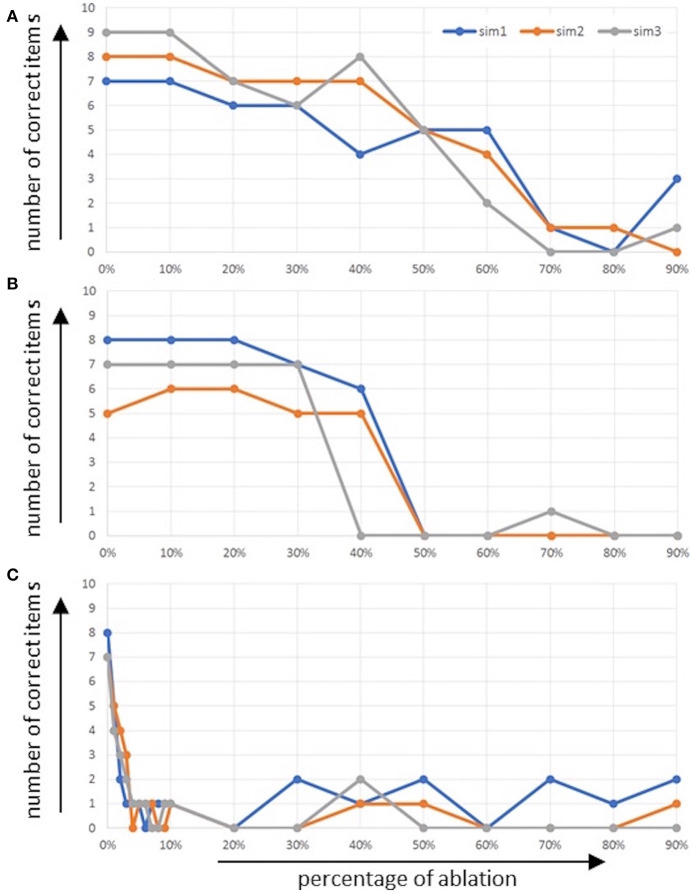
Number of correctly named words (number of correct items) as function of percentage of ablated neurons **(A)** within the concept input buffer and **(B)** within the concept through-to-out clean up associative memory, and **(C)** within the memory buffer.

The overall performance of the model for the repetition of a word list task (i.e., the number of correctly named words) is given in Figure 6 as a function of the percentage of ablated neurons within the respective buffers, i.e., ablation of the concept input buffer (Figure 6A), ablation of the concept through-to-out cleanup associative memory (Figure 6B), as well as ablation of the memory buffer (Figure 6C). It should be kept in mind that an associative memory which connects between two buffers itself is a buffer.

It can be seen that even in the case of the normal model (physiological case, no ablation of neurons) the mean number of words which can be recalled and repeated correctly is about seven words (five to nine words, see Figure 6). In the case of ablation of neurons within the concept input buffer, it can be seen that number of correct words decreases only slightly up to 40% and then decreases down to about one correct word between 40 and 70% ablation (Figure 6A). In the case of the ablation of neurons within the associative cleanup memory between the concept through and concept output buffers, we see comparably good performance up to 30% ablation but then a very fast and strong decay of performance down to one or no correct words at 50% ablation (Figure 6B).

In the case of the ablation of neurons in the memory buffer (mem in Figure 1), the situation is different. Here, the performance of the model directly decreases if only 1% of the neurons in the memory are ablated; the performance of word repetition decreases down to zero or one correctly repeated word at about 4% ablation in this buffer (Figure 6C). This may result from the fact that (1) the memory must hold activation patterns over seconds in order to allow for correct recall, or from the fact that (2) the process of memorizing and recalling words is modeled here using a complex binding and unbinding process.

Figure 5 shows four more cases, as an example of incorrect output. Only the *Conceptual Production Buffer* (Figures 5B–E) is shown as output, since the input is identical to Figure 5A. In the case of 30% ablation in the conceptual input buffer, the concepts still occur correctly but with a very low neural activation (a factor of five lower in comparison to no ablation). It can be seen that the activation of the semantic pointers is still clearly visible and correct here for the ten words and that the activation of these ten words has a clearly separable activation to other semantic pointers. Even after the following clean-up process, the ten words are still processed nearly correctly at this degree of ablation. Thus, even for these low activation levels within the concept input buffer, a clear activation of the bindings of position and concept pointers occurs in the memory buffer, and does not disturb the correct naming of six out of ten words in the sample case displayed in Figure 5B. Higher degrees of ablation (about 70%) are needed in the concept input buffer in order to decrease the performance down to nearly zero.

In the case of the associative cleanup memory between the concept through and concept output buffers in the cognitive processing module, ablation seems to have no strong influence at low percentages. From Figure 5C, it can be seen that at 30% ablation the concept output buffer still shows normal activity similar to the activity of this buffer displayed in Figure 5A (seven out of ten correctly named items). In Figure 5D it can be seen that in the case of 50% ablation, a completely different activation pattern occurs, leading to a non-sense output in the concept production buffer. Only wrong words are activated in concept production buffer in the example displayed in Figure 5D.

In contrast, ablations in the memory buffer have a strong influence on the performance of the model even at low percentages. Figure 5E shows the performance of the model at 3% ablation. The semantic pointer activity of the memory buffer during the RECALL and REPEAT time interval is about a factor of two lower than in the case of no ablation. This leads to a strongly reduced rate of correct word repetition (three out of ten correctly named items; concept production buffer in Figure 5E compared to Figure 5A).

The experimental data from DemTect shows that the mean number of words that can be retrieved and correctly repeated is 6.5 words (Calabrese et al., 2004; Kalbe et al., 2004). The standardized test evaluation of the DemTect results in a full score for 6.5 correctly named items for people under 60 years old, and a full score for 5.5 correctly named in terms for people over 60 years old. A full score means no limitations in memory ability. Starting with 6 correctly named items under the age of 60, there is already suspicion of mild cognitive impairment (5 or less correctly recalled items for over 60s).

## Discussion

A large-scale neural model has been introduced based on the NEF and the SPA frameworks. This model is capable of simulating speech production and speech perception, as well as the cognitive processing involved in two medical screenings: the WWT and the DemTect. It has been demonstrated how a large-scale model should be structured in order to simulate cognitive tasks and speech processing. For this case, the knowledge repository, i.e., the mental lexicon, associates phonological, and conceptual forms of words. The concepts activated from the mental lexicon are input or output of the cognitive processing component. In our approach concepts are realized as semantic pointers and are used as units for linguistic as well as for cognitive processing (cf. Blouw et al., 2016; Crawford et al., 2016). The skill repository which needs to be activated and accessed in the case of associating phonological forms with motor plans or with auditory representations is beyond the scope of this paper but has been focused on in earlier publications using our approach (Bekolay, 2016; Kröger et al., 2016). The semantic and phonological knowledge repository is implemented here as a predefined number of semantic pointers, associating neural activation patterns with phonological forms, lemmata, and concepts via mathematically defined vectors with a pre-defined dimensionality. This is based on the concept of the Semantic Pointer Architecture (SPA, Eliasmith, 2013) in combination with basic ideas of the Neural Engineering Framework (NEF, ibid.). This approach provides an elegant solution to the problem of data processing in a large-scale neural model and has been used in neural models of a wide range of other tasks (Eliasmith, 2012; Choo, 2018).

We argued that it is helpful to simulate well-defined scenarios for the purposes of testing large-scale neural models. Here, parts of two subtests of two different medical screenings have been used as well-defined simulation scenarios. The model was instructed (1) to repeat a word list (part of DemTect, Kalbe et al., 2004) and (2) to name 20 words on the basis of specific visual input (part of WWT, Glück, 2011). Because behavioral data exist for medical screenings, it is possible to check whether the neural model behaves in a “normal human range” or behaves “pathologically.” This allows us to rate the quality of the neural model.

Moreover, specific neural deficits can be introduced into the model that lead to specific deviations in behavior. Deficits are introduced here through the ablation of neurons in specific buffers of the model. Our simulations indicate that ablations like this can have different effects. In the case of a recurrent buffer that is used as a memory and is involved in complex binding and unbinding processes, the ablation of even a small percentage of neurons leads to strong effects in the form of decreased test performance. In other cases, neural ablation can be massive (up to 30% of all neurons of a buffer) before effects occur at the level of model behavior. Thus, the neural model here seems to be relatively robust. The same holds for the associative memories at the conceptual level of word production as well as those for the association between words and lemmata at the end of the cognitive processing component and within the production pathway. Here, behavioral effects like the production of wrong words or no words start to occur if the proportion of ablated neurons goes above roughly 30%.

Even though an increase in the percentage of ablated neurons in a buffer on the output side (production pathway) leads to an abrupt change in model behavior, the same thing does not occur on the input side (ablation in a concept buffer at the procedural beginning of cognitive processing). In this later case (input side), we see a slow decrease, e.g., a more continuous decrease in test performance in the word repetition test. This decrease starts at about 20% ablation and ends at about 70% ablation. These effects of model behavior resulting from ablation of neurons in different modules and different buffers within the neural model need to be investigated in more detail in further studies.

If we compare our simulated data with the experimental data (standard data) of WWT for the corresponding age category (7;6 to 7;11) and nouns (Glück, 2011) the simulation results can be considered as similar. In the simulated physiological case (0% ablation), all 20 items are correctly named in all cases. The results of Glück (2011) also show that a few children in the appropriate age category are able to name all 20 items correctly. This is comparable to a richly filled mental lexicon and intact language processing. But if all children are included (also including children with language disorder), on average 11 of the 20 items were named correctly. Eleven is therefore a cut-off value. If we summarize all simulated results (physiological and pathological), the use of ablation in the concept buffer shows the same effect. Fifty percent of the results are over 11 correctly named items and 50% at or below 11 correctly named items. This shows that the performance of the children in the experimental group is very heterogeneous (as in the simulation results) reflected in the number of correctly named items. These results address the storage hypothesis that they may have acquired fewer words, or have poorly organized conceptual storage (McGregor and Appel, 2002; Gray, 2005; Seiger-Gardner and Schwartz, 2008). This can be seen in poorer naming (Messer and Dockrell, 2013).

A dysfunction in a connection buffer (e.g., the clean-up associative memory between the conceptual and the word production buffer) has less effect on the simulation results in terms of a concept level disturbance. Here 70% of the results are over 11 correctly named items and 30% are at or below 11 correctly named items. This supports the retrieval hypothesis that the mental lexicon is comparable to that of a normally developed child, but lexical processing (i.e., word retrieval) is less efficient (Fried-Oken, 1987; Newman and German, 2002). Intact levels of the mental lexicon seem to compensate longer for a disturbance in the form of ablation.

If we compare our data with the experimental data (standard data) of DemTect (Calabrese et al., 2004; Kalbe et al., 2004) the simulation results can be considered as similar for the physiological case. It can be seen in this case that the mean number of words which can be recalled and repeated correctly is about seven words (five to nine words, see Figure 6). As mentioned, the standardized test evaluation of the DemTect gives a full score for people under 60 with 6.5 correctly named items and 5.5 correctly named items for people over 60. The results with ablation show that a dysfunction in the memory leads to a rapid loss of recall abilities. The both other dysfunctions show in comparison to that a slight decrease. This is also shown by the standardized test evaluations. Starting with six correctly named items (or five, depending on the age category) there is already a suspicion of mild cognitive impairment.

To clarify the nature of our modeling work, some limitations will be discussed below. The present neural computational model is biologically inspired and rooted in known facts about the physiology of speech processing. However, in the model buffers are not assigned to specific regions but they are functionally defined. Nevertheless, we can formulate hypothetical assignments, since the localization of the mental lexicon and language processing in the brain has been researched sufficiently by several imaging studies (for a review see Indefrey and Levelt, 2000, 2004; Indefrey, 2011).

Another limitation concerns learning processes, which are not present in the current model. The neural connection weights are calculated for the defined associations between buffers (see Eliasmith, 2013; Stewart and Eliasmith, 2014). Further, the task control for the individual tasks within the WWT and the DemTect, which is simulated using the basal ganglia thalamus model developed by Stewart et al. (Stewart et al., 2010a,b, 2012a) is pre-defined in a task-specific way. In case of our modeling, only the decision processes required for our two tasks (WWT and DemTect) were implemented which allows our neural model only to simulate the exact tasks.

Furthermore, the input functions are prescriptive: visual and auditory input is only allowed at given time intervals and these inputs are processed within specific time intervals in our model. Thus, the time interval over which certain processing steps are performed is fixed in our simulations (e.g., see PRODUCE_NOMEN in Figure 3).

Finally, the size of the vocabulary is still limited. The vocabulary used in our simulations was created only for the two specific tasks. This vocabulary can be interpreted as a basic vocabulary that is needed by the mental lexicon in order to perform the tasks successfully. However, it should be noted that in the case of WWT, the vocabulary needed to perform the whole WWT (R1-R4) has been included. This means that there are over 1,000 entries. Thus, the size is not representative of an entire vocabulary, but it is still large enough for mistakes to arise.

## Conclusions

A first long-term goal of our research is to discover the underlying neural defects which cause specific behavioral deficits as quantified in medical screenings. This association between neural defects in specific modules or neural buffers of a large-scale neural model and behavioral deficits displayed by the model simulating a medical screening is important because it allows us to associate neural deficits with specific disorders and impairments. Our neural model provides a hypothesis concerning which neural deficits or neural dysfunctions lead to which cognitive or speech and language disorders or impairments. Thus, our modeling work can be used as a research tool to associate behavioral deficits, dysfunctions or disorders with neural deficits or dysfunctions introduced within the model.

A second long-term goal of our research is to use neural models and the resulting simulations in order to optimize the sensitivity of screenings with respect to specific cognitive, speech, and language disorders. This goal can be reached by varying specific parameters of a screening (e.g., the length of a word list, the selection of different types of test words, the selection of different subtasks for a screening, etc.) for a defined “pathological” neural model. Thus, neural models and computer simulations might make it possible to check which modifications of a screening are most effective for detecting a specific disorder or impairment. Thus, our modeling and simulation tool is able to check the change of sensitivity of a screening with respect to a variation of different screening parameters. This goal is difficult to reach if exclusively natural data are inspected because without using neural models a huge number of subjects needs to be tested using different versions of a screening.

## Author Contributions

BK, TB, PB, and CS contributed to software coding. BK and CS conducted the simulation experiments. All authors contributed to planning the study and writing and correcting the manuscript.

### Conflict of Interest Statement

TB and PB are employed by Applied Brain Research, Inc., Waterloo, Canada. The remaining authors declare that the research was conducted in the absence of any commercial or financial relationships that could be construed as a potential conflict of interest.
